# HIV indirectly accelerates coronary artery disease by promoting the effects of risk factors: longitudinal observational study

**DOI:** 10.1038/s41598-021-02556-w

**Published:** 2021-11-30

**Authors:** Márton Kolossváry, David Celentano, Gary Gerstenblith, David A. Bluemke, Raul N. Mandler, Elliot K. Fishman, Sandeepan Bhatia, Shaoguang Chen, Shenghan Lai, Hong Lai

**Affiliations:** 1 Department of Pathology, Johns Hopkins University School of Medicine, 600 N Wolfe St, Pathology #301, Baltimore, MD 21287 USA; 2 MTA-SE Cardiovascular Imaging Research Group, Heart and Vascular Center, Semmelweis University, 68 Városmajor str., Budapest, Hungary 1122; 3 Department of Epidemiology, Johns Hopkins University Bloomberg School of Public Health, 614 Wolfe N Wolfe St, Baltimore, MD 21205 USA; 4 Department of Medicine, Johns Hopkins University School of Medicine, 733 N Broadway, Baltimore, MD 21205 USA; 5 School of Medicine and Public Health, University of Wisconsin, 750 Highland Ave, Madison, WI 53726 USA; 6 National Institute on Drug Abuse, National Institutes of Health, 10 Center Dr, Bethesda, MD 20814 USA; 7 Department of Radiology, Johns Hopkins University School of Medicine, 601 N Caroline St, Baltimore, MD 21205 USA; 8 Institute of Human Virology, University of Maryland School of Medicine, 725 W Lombard St, Baltimore, MD 21201 USA

**Keywords:** Diseases, Medical research

## Abstract

Our objective was to assess whether human immunodeficiency virus (HIV)-infection directly or indirectly promotes the progression of clinical characteristics of coronary artery disease (CAD). 300 African Americans with asymptomatic CAD (210 male; age: 48.0 ± 7.2 years; 226 HIV-infected) who underwent coronary CT angiography at two time points (mean follow-up: 4.0 ± 2.3 years) were randomly selected from 1429 participants of a prospective epidemiological study between May 2004 and August 2015. We calculated Agatston-scores, number of coronary plaques and segment stenosis score (SSS). Linear mixed models were used to assess the effects of HIV-infection, atherosclerotic cardiovascular disease (ASCVD) risk, years of cocaine use on CAD. There was no significant difference in annual progression rates between HIV-infected and—uninfected regarding Agatston-scores (10.8 ± 25.1/year vs. 7.2 ± 17.8/year, *p* = 0.17), the number of plaques (0.2 ± 0.3/year vs. 0.3 ± 0.5/year, *p* = 0.11) or SSS (0.5 ± 0.8/year vs. 0.5 ± 1.3/year, *p* = 0.96). Multivariately, HIV-infection was not associated with Agatston-scores (8.3, CI: [− 37.2–53.7], *p* = 0.72), the number of coronary plaques (− 0.1, CI: [− 0.5–0.4], *p* = 0.73) or SSS (− 0.1, CI: [− 1.0–0.8], *p* = 0.84). ASCVD risk scores and years of cocaine-use significantly increased all CAD outcomes among HIV-infected individuals, but not among HIV-uninfected. Importantly, none of the HIV-medications were associated with any of the CAD outcomes. HIV-infection is not directly associated with CAD and therefore HIV-infected are not destined to have worse CAD profiles. However, HIV-infection may indirectly promote CAD progression as risk factors may have a more prominent role in the acceleration of CAD in these patients.

## Introduction

Antiretroviral therapy (ART) has transformed the disease course of human immunodeficiency virus (HIV)-infection. While manifestations of acquired immunodeficiency syndrome are now scarce, people living with HIV (PLWH) still have shorter life expectancies as compared to the general population due to the increased risk of noncommunicable diseases such as coronary artery disease (CAD)^1,2^. While data on hard end-points (myocardial infarction and death) show clear associations with HIV-infection, information regarding sub-clinical stages of CAD are contradictory^3–6^. It is unclear whether discrepancies of previous results are due to the cross-sectional study design, or whether there may be other factors which promote CAD in PLWH, which were less investigated, such as illegal substance use. However, the chronic effects of substance use (such as cocaine) on CAD also prove to be controversial with results showing clear associations^5,7^, while other investigations failed to identify any connection^8,9^. As nearly half of all PLWH report substance use beyond marihuana^10,11^, it is of utmost importance to investigate how conventional (cardiovascular) and unconventional (HIV-infection and substance abuse) risk factors accelerate subclinical CAD in PLWH.

Although African Americans constitute only about 13% of the U.S. population, 43% of the estimated new cases of HIV in 2016 were in African Americans^12^. Among reasons for the high prevalence of HIV-infection among African Americans is drug abuse, the second leading cause of HIV-infection in both African Americans men and women^12^. Thus, it is crutial to understand the interplay of risk factors and CAD in this patient population.

Coronary CT angiography (CTA) provides a non-invasive method to assess CAD. Coronary atherosclerosis may be characterized by the Agatston-score, an independent predictor of future major adverse cardiac events^13^. However, Agatston-score does not detect noncalcified plaques which are strong predictors of later outcomes^14^. Furthermore, it is not only the number of plaques, but the extent of CAD and the degree of stenosis, which are important and which can be summarized by the segment stenosis score (SSS)^15^.

Therefore in our prospective longitudinal observational study, we wished to assess whether HIV-infection had an independent effect on CAD or whether it may facilitate atherosclerosis through promoting the effects of risk factors in African Americans. Furthermore, we wished to assess the potential adverse effects of illicit drugs in this patient population.

## Materials and methods

Detailed study methodologies can be found in supplementary material.

### Study design and participants

Between May 2004 and August 2015, 1429 cardiovascularily asymptomatic individuals were prospectively enrolled in an epidemiological observational study investigating the effects of illicit drugs, HIV, ART and inflammation on CAD^5,7^. Inclusion criteria were (1) age ≥ 21 years and (2) self‐designated African American race. Exclusion criteria were: (1) history of serious physical disease or current physical disease which may preclude the follow-up of patients or hinder the image quality of coronary CT angiography, including chronic obstructive pulmonary disease; (2) pregnancy; (3) chronic kidney disease with an estimated glomerular filtration rate of < 60 mL/minute per 1.73 m^2^; and (4) contraindication to CT angiography scans, including a history of contrast allergy.

Previously, in this cohort the associations between HIV-infection and clinical characteristics of CAD (presence of CAD and presence of different plaque types) were investigated based-on cross-sectional data^5^. In the current manuscript we aimed to assess the longitudinal effect of illicit drugs on coronary Agatston-score, number of coronary plaques present and SSS. With the intention of later volumetric analysis we deemed the analysis of 300 individuals’ 2 CT scans feasible and comparable to other CAD progression analyses in different patient populations^16^. Therefore, we randomly selected 300 individuals showing at least > 10% stenosis (definition of subclinical CAD) on the baseline coronary CTA^17^.

### Demographic characteristics

Briefly, all participants self-reported years of cocaine use defined as use by any route for at least 6 months, administered at least 4 times a month^5^. Atherosclerotic cardiovascular disease (ASCVD) risk scores were calculated as a measure of overall cardiovascular risk based on the ACC/AHA Guidelines at the time of the CTA scans^18^. All participants were tested for HIV-infection by ELISA and if positive, then confirmed by Western blot test. Furthermore, at the time of the CTA examinations high‐sensitivity C‐reactive protein (hsCRP) was also measured. For HIV‐infected participants, detailed information regarding duration of known HIV-infection, and medications including: nucleoside reverse-transcriptase inhibitor use (NRTI), non-nucleoside reverse-transcriptase inhibitor use (NNRTI) and protease inhibitor use (PI) were collected. A medical chart review was used to confirm the information on the medical history and medications provided by the study participants.

### CAD characteristics

Detailed scan protocols have been published^5,7^. Briefly, coronary Agatston-score was calculated using the Agatston-score method^19^. Coronary plaque was defined as any discernible structure that could be assigned to the coronary artery wall and could be identified in at least 2 independent planes and caused at least a 10% reduction in lumen caliber^17^. Overall, the total number of plaques present in the coronaries was counted. Furthermore, the degree of stenosis present in each of 17 coronary segments was classified as: (0) none (0%); (1) minimal (10–24%); (2) mild (25–49%); (3) moderate (50–69%), (4) severe (70–99%) or (5) occluded (100%) and used to quantify the SSS by summing the stenosis score of each segment per patient^20^. All images were randomly interpreted by a level-3 certified specialist in cardiac CT imaging with 5 years of experience and blinded to all clinical data.

### Statistical analysis

In brief, we used linear mixed models which allow the analysis of repeated observations at non-standardized intervals. It also allows to assess whether a risk factors affects the overall amount of an outcome (i.e. Agatston-score) and/or if it changes the annual progression rate of the outcome (i.e. yearly progression of Agatston-score)^21,22^. We calculated univariate models to assess the effect of each predictor. If it was associated with the overall amount or the progression rate of the given outcome at *p* < 0.10, then we included that predictor into a multivariate model. We always included HIV in the multivariate models as we wished to assess its potential modifying effect. We further did sub-analyses stratifying our data based-on HIV-serostatus to assess whether HIV modifies the effect of cocaine use. For HIV positive cohorts, we also included HIV-associated medications in the analysis. We also stratified our patient population to cocaine-users: individuals who used cocaine at either time point; and cocaine non-users: participants who did not use cocaine at either time point to remove the potential effects of cocaine use on outcomes. All analyses were conducted in the R environment v3.6.1^23^. A 2-sided *p* value smaller than 0.05 was considered statistically significant.

### Ethics approval

The Institutional Review Board at the Johns Hopkins School of Medicine (Baltimore, MD) approved the study protocol. All procedures used in this study were in accordance with HIPAA, local and federal regulations, and the Declaration of Helsinki.

### Consent to participate

All study participants provided written informed consent.

## Results

### Characteristics and cardiovascular risk profiles of the participants

A total of 300 study participants were included in this investigation. The demographic and clinical characteristics of all participants and HIV‐related clinical factors of HIV‐infected participants are presented in Table 1.Of the 300 participants, 210 (70.0%) were men and the mean age was 48.0 ± 7.2 years and 226 (75.3%) were infected with HIV at baseline, among HIV-infected, the mean CD4 count and log viral load were 446 ± 283 cell/mm^3^ and 2.4 ± 1.1 copies/mL, respectively. During the follow-up period, none of the HIV-uninfected participants acquired HIV. The reported cocaine use increased from 161 to 174 over the follow-up period (*p* = 0.29). The average duration of cocaine use among cocaine users increased from 13.7 ± 8.7 years at baseline to 14.9 ± 8.6 years at the end of follow-up (*p* < 0.0001). The mean ASCVD risk score increased from 7.6% ± 7.2% at baseline to 10.2% ± 7.8% (*p* < 0.0001) between the two CT scans (4.0 ± 2.3 years).

**Table 1:** Demographic, behavioral, clinical and laboratory characteristics at baseline and follow-up stratified by HIV-serostatus.

Characteristic	HIV-infected (n = 226)			HIV-uninfected (n = 74)			HIV-infected vs. HIV-uninfected	
	Baseline	Follow-up	*p*	Baseline	Follow-up	*p*	Baseline *p*	Follow-up *p*
**Anthropometrics**								
Age (year)	48.7 ± 7.0	52.7 ± 7.2	< 0.0001	45.8 ± 7.6	49.7 ± 7.3	< 0.0001	0.405	0.570
Male sex (n, %)	163 (72.1%)	163 (72.1%)	1.000	47 (63.5%)	47 (63.5%)	1.000	0.188	0.188
BMI (kg/m^2^)	26.2 ± 5.1	26.7 ± 5.5	0.010	27.7 ± 5.7	27.5 ± 5.5	0.711	0.056	0.068
Follow-up time (years)		4.0 ± 2.2			3.8 ± 2.6			0.416
**Cardiovascular risk factors**								
ASCVD risk (%)	8.1 ± 7.7	10.7 ± 8.0	< 0.0001	6.0 ± 5.0	8.5 ± 7.1	< 0.0001	0.814	0.708
Hypertension (n, %)	33 (14.6%)	67 (29.7%)	< 0.001	10 (13.5%)	22 (29.7%)	0.001	1.000	1.000
Diabetes (n, %)	7 (3.1%)	18 (8.0%)	0.003	2 (2.7%)	3 (4.1%)	1.000	1.000	0.306
Positive family history (n, %)	61 (27.0%)	79 (35.0%)	< 0.0001	18 (24.3%)	24 (32.4%)	0.041	0.761	0.778
Report of cigarette use (n, %)	187 (82.7%)	189 (83.6%)	0.480	61 (82.4%)	62 (83.8%)	1.000	1.000	1.000
Report of alcohol use (n, %)	189 (83.6%)	204 (90.3%)	0.001	66 (89.2%)	67 (90.5%)	1.000	0.348	1.000
Report of cocaine use (n, %)	111 (49.1%)	124 (54.9%)	< 0.001	50 (67.6%)	50 (67.6%)	1.000	0.007	0.059
Duration of cocaine use among users (year)	15.4 ± 9.0	16.4 ± 9.0	< 0.0001	12.4 ± 7.8	14.7 ± 8.5	< 0.0001	0.423	0.488
Statin users (n, %)	25 (11.1%)	31 (13.7%)	0.041	0 (0.0%)	0 (0.0%)	1.000	< 0.001	< 0.0001
**Lipid profiles and laboratory results**								
Total cholesterol (mg/dL)	169.7 ± 39.9	170.0 ± 39.40	0.916	187.2 ± 34.4	179.7 ± 36.7	0.064	0.768	0.795
LDL-C (mg/dL)	90.6 ± 35.8	88.3 ± 31.1	0.213	108.6 ± 32.0	98.6 ± 33.5	0.010	0.941	0.522
HDL-C (mg/dL)	52.3 ± 18.0	54.8 ± 19.9	0.010	57.9 ± 19.4	61.2 ± 25.7	0.076	0.216	0.202
Triglycerides (mg/dL)	131.7 ± 81.8	139.6 ± 93.0	0.207	103.3 ± 57.3	106.7 ± 54.6	0.826	0.092	0.854
Fasting glucose (mg/dL)	88.5 ± 20.4	95.5 ± 45.4	0.009	93.6 ± 45.4	94.8 ± 36.7	0.686	0.237	0.504
hsCRP (mg/dL)	3.1 ± 4.6	4.7 ± 9.9	0.015	3.8 ± 5.5	5.5 ± 11.3	0.185	0.052	0.079
**HIV associated factors**								
Time since HIV diagnosis (year)	21.7 ± 8.8	25.7 ± 9.3	< 0.0001					
Antiretroviral therapy users (n, %)	200 (91.3%)	207 (95.8%)	0.013					
NRTI users (n, %)	190 (86.8%)	197 (91.2%)	0.013					
Duration of NRTI use (year)	5.7 ± 5.3	8.3 ± 6.2	< 0.0001					
NNRTI users (n, %)	100 (45.7%)	110 (50.9%)	0.004					
Duration of NNRTI use (year)	5.1 ± 4.8	6.9 ± 5.4	< 0.0001					
PI users (n, %)	158 (72.2%)	166 (76.9%)	0.008					
Duration of PI use (year)	6.1 ± 7.1	8.5 ± 7.7	< 0.0001					
**Visual characteristics of coronary artery disease**								
Agatston-score (unit)	56.4 ± 195.0	102.6 ± 279.8	< 0.0001	35.2 ± 82·0	64.8 ± 163.2	0.013	0.088	0.114
Number of plaques (unit)	2.1 ± 2.0	2.9 ± 2.1	< 0.0001	1.9 ± 1·9	2.8 ± 2.0	< 0.0001	0.888	0.736
Segment Stenosis Score (unit)	3.1 ± 3.6	5.0 ± 5.0	< 0.0001	2.8 ± 3·1	4.1 ± 4.0	< 0.0001	0.411	0.330

Average and standard deviation for continuous variables, frequencies and proportion (%) for categorical variables are reported. *p* values are based on t-test or paired t-test as appropriate or chi-square test or McNemar-test as appropriate.

Abbreviations: *ASCVD risk* cardiovascular risk defined by the ACC/AHA Guideline on the Assessment of Cardiovascular Risk; *BMI* body mass index (kg/m^2^); *LDL-C* low density lipoprotein cholesterol; *HDL-C* high density lipoprotein cholesterol; *HIV* human immunodeficiency virus; *hsCRP* high‐sensitivity C‐reactive protein; *NRTI* nucleoside reverse-transcriptase inhibitors; *NNRTI* non-nucleoside reverse-transcriptase inhibitors; *PI* protease inhibitors.

Reused from: Kolossváry et al. Eur Radiol . 2021 Mar 3. https://doi.org/10.1007/s00330-021-07755-7. Online ahead of print.

### CAD characteristics in the total population

At baseline 118 (39.3%), while at follow-up 144 (48.0%) participants had Agatston-scores greater than zero (*p* = 0.04). The mean Agatston-score changed from: 51.2 ± 174.6 to 93.3 ± 256.8 over the follow-up period (*p* < 0.0001). At baseline there were 621 plaques, on average 2.1 ± 2.0 per patient, were visible, while at follow-up 861 plaques, on average 2.9 ± 2.1, were identified (*p* < 0.0001). Overall, the mean annual change in Agatston-score was 9.9 ± 23.6 units. On average, 0.26 ± 0.34 plaques developed each year and SSS increased by 0.54 ± 0.97 annually.

### Effect of risk factors on CAD parameters—total population

In multivariate analysis, HIV-infection was not associated with either CAD parameter (*p* > 0.05 for all). However, each year of cocaine use increased Agatston-scores by 3.36 units (CI: [1.44–5.27], *p* = 0.001), the number of coronary plaques by 0.03 (CI: [0.01–0.05], *p* = 0.014) and SSS by 0.05 (CI: [0.01–0.09], *p* = 0.012). Furthermore, each percentage increase in ASCVD risk score increased the annual progression rate of Agatston-score by 0.88 units/year (CI: [0.52–1.24], *p* < 0.0001), and the overall number of coronary plaques by 0.03 (CI: [0.01–0.04], *p* = 0.001) and SSS by 0.06 (CI: [0.02–0.09], *p* = 0.002). Also, on average statin users had more disease regarding all three CAD markers as compared to individuals who did not take statins. Detailed univariate and multivariate results are presented in Table 2.

**Table 2:** Predictors of coronary artery disease outcomes among the total population.

Outcome	Predictor	Univariate models						Multivariate models					
		Overall effect on outcome			Effect on progression rate			Overall effect on outcome			Effect on progression rate		
		β	95% CI	*p*	β	95% CI	*p*	β	95% CI	*p*	β	95% CI	*p*
Agatston-score (unit)	HIV*	21.14	[− 24.58–66.87]	0.366	3.64	[− 2.55–9.82]	0.250	8.26	[− 37.20–53.65]	0.721	0.48	[− 5.16–6.11]	0.868
	ASCVD (%)	0.59	[− 0.48–1.67]	0.271	**1.06**	**[0.70**–**1.41]**	** < 0.0001**	0.56	[− 0.47–1.60]	0.282	**0.88**	**[0.52**–**1.24]**	** < 0.0001**
	Cocaine use (y)	**3.51**	**[1.59**–**5.43]**	** < 0.001**	**0.39**	**[0.12**–**0.65]**	**0.005**	**3.36**	**[1.44**–**5.27]**	**0.001**	0.15	[− 0.09–0.40]	0.224
	Positive family history	− 3.57	[− 27.47–20.35]	0.770	**8.31**	**[2.47**–**14.14]**	**0.006**	2.27	[− 19.89–24.41]	0.840	4.54	[− 0.81–9.91]	0.096
	Statin use	**87.8**	**[44.27**–**131.36]**	** < 0.0001**	8.66	[− 0.05–17.40]	0.053	**74.05**	**[32.32**–**115.53]**	** < 0.0001**	5.49	[− 2.85–13.88]	0.197
	hsCRP (mg/dL)	0.26	[− 0.76–1.27]	0.621	− 0.14	[− 0.61–0.33]	0.554						
Number of coronary plaques (unit)	HIV*	0.09	[− 0.43–0.61]	0.741	0.02	[− 0.05–0.08]	0.613	− 0.09	[− 0.58–0.41]	0.731	0.01	[− 0.05–0.08]	0.686
	ASCVD (%)	**0.03**	**[0.01**–**0.05]**	** < 0.001**	0.00	[− 0.01–0.00]	0.142	**0.03**	**[0.01**–**0.04]**	**0.001**	0.00	[− 0.01–0.00]	0.207
	Cocaine use (y)	**0.03**	**[0.01**–**0.05]**	**0.008**	0.00	[0.00–0.00]	0.535	**0.03**	**[0.01**–**0.05]**	**0.014**	0.00	[0.00–0.00]	0.358
	Positive family history	**0.41**	**[0.08**–**0.74]**	**0.016**	− 0.04	[− 0.10–0.02]	0.199	**0.35**	**[0.02**–**0.67]**	**0.035**	− 0.05	[− 0.11–0.01]	0.109
	Statin use	**1.07**	**[0.49**–**1.66]**	** < 0.001**	− 0.03	[− 0.11–0.05]	0.463	**0.97**	**[0.40**–**1.55]**	**0.001**	− 0.01	[− 0.09–0.07]	0.849
	hsCRP (mg/dL)	**0.03**	**[0.01**–**0.04]**	** < 0.001**	− **0.01**	**[**− **0.01**–**0.00]**	**0.007**	**0.02**	**[0.01**–**0.04]**	**0.001**	− **0.01**	**[**− **0.01**–**0.00]**	**0.007**
Segment Stenosis Score (unit)	HIV*	0.25	[− 0.72–1.23]	0.612	0.17	[− 0.02–0.35]	0.074	− 0.10	[− 1.01–0.81]	0.835	0.09	[− 0.09–0.26]	0.338
	ASCVD (%)	**0.07**	**[0.03**–**0.10]**	** < 0.001**	**0.01**	**[0.00**–**0.02]**	**0.024**	**0.06**	**[0.02**–**0.09]**	**0.002**	0.01	[0.00–0.02]	0.114
	Cocaine use (y)	**0.06**	**[0.02**–**0.10]**	**0.008**	**0.01**	**[0.00**–**0.02]**	**0.021**	**0.05**	**[0.01**–**0.09]**	**0.012**	0.01	[0.00–0.01]	0.102
	Positive family history	**0.94**	**[0.19**–**1.68]**	**0.014**	0.04	[− 0.14–0.21]	0.688	**0.70**	**[0.01**–**1.39]**	**0.046**	− 0.07	[− 0.23–0.10]	0.435
	Statin use	**2.62**	**[1.36**–**3.87]**	** < 0.0001**	0.22	[− 0.01–0.46]	0.063	**2.35**	**[1.15**–**3.54]**	** < 0.0001**	0.20	[− 0.04–0.43]	0.100
	hsCRP (mg/dL)	**0.09**	**[0.05**–**0.13]**	** < 0.0001**	− 0.01	[− 0.02–0.00]	0.122	**0.09**	**[0.06**–**0.13]**	** < 0.0001**	− **0.02**	**[**− **0.03**–**0.00]**	**0.021**

*: HIV was forced into the multivariate model.

Abbreviations: *β* unstandardized beta coefficients from linear mixed model; *ASCVD risk* cardiovascular risk defined by the ACC/AHA Guideline on the Assessment of Cardiovascular Risk; *CI* confidence interval; *HIV* human immunodeficiency virus; hsCRP: high‐sensitivity C‐reactive protein.

*p* values smaller than 0.05 are indicated in bold.

### Stratification analysis based-on HIV serostatus

There was no significant difference in any of the CAD parameters between PLWH and uninfected, neither at baseline, nor at follow-up (Table 1).

There was no significant difference in the annual progression rates of any CAD parameter between PLWH and uninfected participants (supplemental Table 1). However, as multiple factors may play a role in the acceleration of CAD and population-based assessments are less powerful than analyzing the intra-individual changes in parameters, we conducted multivariate analyses using linear mixed models.

Among PLWH, in multivariate models, each year of cocaine use increased Agatston-scores by 4.05 units (CI: [1.67–6.43], *p* = 0.001), the number of coronary plaques by 0.03 (CI: [0.00–0.05], *p* = 0.020) and SSS by 0.07 (CI: [0.02–0.11], *p* = 0.005). Each percentage increase in ASCVD risk scores increased the annual progression rate of Agatston-scores by 0.87 units/year (CI: [0.46–1.27], *p* < 0.0001), the overall number of coronary plaques by 0.02 (CI: [0.01–0.04], *p* = 0.001) and SSS by 0.05 (CI: [0.02–0.09], *p* = 0.004). Importantly, none of the HIV associated medications had effect on any of the CAD parameters. Detailed results are presented in Table 3*.*

**Table 3:** Predictors of coronary artery disease outcomes among people living with HIV.

Outcome	Predictor	Univariate models						Multivariate models					
		Overall effect on outcome			Effect on progression rate			Overall effect on outcome			Effect on progression rate		
		β	95% CI	*p*	β	95% CI	*p*	β	95% CI	*p*	β	95% CI	*p*
Agatston-score (unit)	ASCVD (%)	1.12	[− 0.13–2.38]	0.080	**1.06**	**[0.64**–**1.48]**	** < 0.0001**	1.08	[− 0.10–2.28]	0.073	**0.87**	**[0.46**–**1.27]**	** < 0.0001**
	Cocaine use (y)	**4.17**	**[1.76**–**6.57]**	** < 0.001**	**0.49**	**[0.17**–**0.81]**	**0.003**	**4.05**	**[1.67**–**6.43]**	**0.001**	0.25	[− 0.04–0.54]	0.095
	Positive family history	− 2.76	[− 33.36–27.84]	0.860	**10.16**	**[3.05**–**17.27]**	**0.006**	3.98	[− 23.76–31.75]	0.778	5.68	[− 0.77–12.13]	0.084
	Statin use	**90.74**	**[43.42**–**138.05]**	** < 0.001**	7.99	[− 1.41–17.43]	0.098	**73.56**	**[28.98**–**117.75]**	**0.001**	5.11	[− 3.70–13.98]	0.256
	hsCRP (mg/dL)	− 0.13	[− 1.73–1.47]	0.874	− 0.11	[− 0.72–0.50]	0.732						
	PI use (y)	2.73	[− 0.76–6.17]	0.121	0.37	[− 0.08–0.82]	0.110						
	NRTI use (y)	2.12	[− 2.06–6.24]	0.306	0.41	[− 0.14–0.95]	0.144						
	NNRTI use (y)	− 1.16	[− 6.68–4.30]	0.677	− 0.13	[− 0.86–0.60]	0.729						
Number of coronary plaques (unit)	ASCVD (%)	**0.03**	**[0.01**–**0.04]**	** < 0.001**	0.00	[− 0.01–0.00]	0.169	**0.02**	**[0.01**–**0.04]**	**0.001**	0.00	[− 0.01–0.00]	0.093
	Cocaine use (y)	**0.03**	**[0.01**–**0.06]**	**0.013**	0.00	[0.00–0.00]	0.938	**0.03**	**[0.00**–**0.05]**	**0.020**	0.00	[0.00–0.00]	0.953
	Positive family history	0.23	[− 0.13–0.59]	0.217	− 0.01	[− 0.07–0.06]	0.844						
	Statin use	**1.03**	**[0.47**–**1.59]**	** < 0.001**	− 0.04	[− 0.13–0.04]	0.296	**1.03**	**[0.49**–**1.58]**	** < 0.0001**	− 0.02	[− 0.11–0.06]	0.599
	hsCRP (mg/dL)	0.01	[0.00–0.03]	0.141	0.00	[− 0.01–0.00]	0.150						
	PI use (y)	**0.04**	**[0.00**–**0.07]**	**0.041**	0.00	[− 0.01–0.00]	0.287	0.02	[− 0.02–0.06]	0.272	0.00	[− 0.01–0.00]	0.853
	NRTI use (y)	0.04	[0.00–0.08]	0.059	− **0.01**	**[**− **0.01**–**0.00]**	**0.018**	0.02	[− 0.03–0.07]	0.437	− 0.01	[− 0.01–0.00]	0.057
	NNRTI use (y)	0.05	[− 0.01–0.10]	0.107	− **0.01**	**[**− **0.01**–**0.00]**	**0.029**	0.03	[− 0.03–0.09]	0.270	− 0.01	[− 0.01–0.00]	0.143
Segment Stenosis Score (unit)	ASCVD (%)	**0.06**	**[0.02**–**0.10]**	**0.002**	**0.02**	**[0.00**–**0.03]**	**0.012**	**0.05**	**[0.02**–**0.09]**	**0.004**	0.01	[0.00–0.02]	0.053
	Cocaine use (y)	**0.07**	**[0.02**–**0.12]**	**0.007**	**0.01**	**[0.00**–**0.02]**	**0.028**	**0.07**	**[0.02**–**0.11]**	**0.005**	0.01	[0.00–0.01]	0.177
	Positive family history	0.83	[− 0.01–1.67]	0.054	0.14	[− 0.06–0.34]	0.183	0.67	[− 0.12–1.47]	0.095	0.02	[− 0.18–0.21]	0.876
	Statin use	**2.80**	**[1.55**–**4.04]**	** < 0.0001**	0.19	[− 0.06–0.44]	0.132	2.48	[1.29–3.68]	** < 0.0001**	0.17	[− 0.07–0.42]	0.165
	hsCRP (mg/dL)	**0.05**	**[0.00**–**0.09]**	**0.047**	− 0.01	[− 0.02–0.01]	0.518	0.04	[0.00–0.08]	0.072	− 0.01	[− 0.02–0.01]	0.318
	PI use (y)	0.07	[− 0.01–0.14]	0.075	0.00	[− 0.01–0.01]	0.821	0.06	[0.00–0.13]	0.063	− 0.01	[− 0.02–0.00]	0.239
	NRTI use (y)	0.06	[− 0.03–0.14]	0.218	− 0.01	[− 0.02–0.01]	0.495						
	NNRTI use (y)	0.08	[− 0.04–0.19]	0.196	− 0.01	[− 0.03–0.01]	0.405						

Abbreviations: *β* unstandardized beta coefficients from linear mixed model; *ASCVD risk* cardiovascular risk defined by the ACC/AHA Guideline on the Assessment of Cardiovascular Risk; *CI* confidence interval; *HIV* human immunodeficiency virus; *hsCRP* high‐sensitivity C‐reactive protein; *NRTI* nucleoside reverse-transcriptase inhibitors; *NNRTI* non-nucleoside reverse-transcriptase inhibitors; PI: protease inhibitors.

*p* values smaller than 0.05 are indicated in bold.

However, among HIV-uninfected, cocaine use had no effect on any of the CAD markers (Table 4). Nevertheless, ASCVD risk score increased the annual progression rate of Agatston-score by 1.38 units/year (CI: [0.74–1.96], *p* < 0.0001), but not any of the other CAD markers. Interestingly, hsCRP had a more prominent effect among HIV-uninfected, by increasing the number of coronary plaques by 0.05 (CI: [0.02–0.08], *p* = 0.001) and SSS by 0.14 (CI: [0.09–0.20], *p* < 0.0001). Detailed results are presented in Table 4.

**Table 4:** Predictors of coronary artery disease outcomes among HIV-uninfected individuals.

Outcome	Predictor	Univariate models						Multivariate models					
		Overall effect on outcome			Effect on progression rate			Overall effect on outcome			Effect on progression rate		
		β	95% CI	*p*	β	95% CI	*p*	β	95% CI	*p*	β	95% CI	*p*
Agatston-score (unit)	ASCVD (%)	− 1.00	[− 3.06–1.11]	0.330	**1.38**	**[0.74**–**1.96]**	** < 0.0001**	− 1.00	[− 3.06–1.11]	0.330	**1.38**	**[0.74**–**1.96]**	** < 0.0001**
	Cocaine use (y)	0.82	[− 1.31–2.98]	0.456	0.12	[− 0.31–0.53]	0.579						
	Positive family history	4.64	[− 24.90–34.06]	0.757	0.75	[− 8.33–9.98]	0.871						
	hsCRP (mg/dL)	0.68	[− 0.53–1.89]	0.264	− 0.10	[− 0.84–0.63]	0.793						
Number of coronary plaques (unit)	ASCVD (%)	0.06	[0.00–0.11]	0.054	0.00	[− 0.01–0.01]	0.461	0.05	[− 0.01–0.10]	0.077	0.00	[− 0.01–0.01]	0.593
	Cocaine use (y)	0.02	[− 0.02–0.07]	0.354	0.00	[0.00–0.01]	0.321						
	Positive family history	**0.76**	**[0.04**–**1.48]**	**0.041**	− 0.08	[− 0.21–0.04]	0.164	**0.72**	**[0.02**–**1.42]**	**0.043**	− 0.06	[− 0.19–0.06]	0.340
	hsCRP (mg/dL)	**0.04**	**[0.01**–**0.07]**	**0.004**	− 0.01	[− 0.02–0.00]	0.073	**0.05**	**[0.02**–**0.08]**	**0.001**	− **0.01**	**[**− **0.03**–**0.00]**	**0.025**
Segment Stenosis Score (unit)	ASCVD (%)	0.08	[− 0.04–0.19]	0.172	0.01	[− 0.01–0.02]	0.556						
	Cocaine use (y)	0.03	[− 0.06–0.12]	0.526	0.01	[− 0.01–0.02]	0.269						
	Positive family history	1.05	[− 0.40–2.51]	0.160	− 0.17	[− 0.45–0.12]	0.258						
	hsCRP (mg/dL)	**0.14**	**[0.09**–**0.20]**	** < 0.0001**	− 0.01	[− 0.04–0.01]	0.418	**0.14**	**[0.09**–**0.20]**	** < 0.0001**	− 0.01	[− 0.04–0.01]	0.418

Abbreviations: *β* unstandardized beta coefficients from linear mixed model; *ASCVD risk* cardiovascular risk defined by the ACC/AHA Guideline on the Assessment of Cardiovascular Risk; *CI* confidence interval; *HIV* human immunodeficiency virus; *hsCRP* high‐sensitivity C‐reactive protein.

*p* values smaller than 0.05 are indicated in bold.

### Stratification analysis based-on cocaine use

Cocaine users tended to have worse CAD parameters as compared to non-users (supplemental Table 2). We found significantly accelerated CAD progression in cocaine-users as compared to non-users with regards to the annual progression rate of Agatston-score and SSS (12.62 ± 24.86/year vs. 6.21 ± 21.19/year, *p* = 0.017; 0.64 ± 1.08/year vs. 0.39 ± 0.76/year, *p* = 0.017, respectively), with borderline differences regarding the annual progression are of the number of plaques (0.28 ± 0.38/year vs. 0.22 ± 0.28/year, *p* = 0.087).

Multivariate analysis indicated, that among cocaine users, HIV-infection had no effect on any of the CAD parameters (*p* > 0.05 for all). However, each percentage increase in ASCVD risk scores increased the number of coronary plaques by 0.06 (CI: [0.03–0.09], *p* < 0.0001) and SSS by 0.12 (CI: [0.05–0.18], *p* < 0.0001). Detailed results can be found in supplemental Table 3.

Among the 126 (42.0%) of cocaine non-users, in multivariate models, HIV-infection had no effect on any CAD marker (*p* > 0.05 for all). However, each percentage increase in ASCVD risk scores increased Agatston-scores by 1.21 units (CI: [0.25–2.16], *p* = 0.012), the number of coronary plaques by 0.02 (CI: [0.00–0.04], *p* = 0.016) and the annual progression rate of Agatston-scores by 1.07 units/year (CI: [0.64–1.50], *p* = 0.012) and SSS by 0.01/year (CI: [0.00–0.03], *p* = 0.036). Detailed results are in supplemental Table 4.

## Discussion

The findings of this investigation show that (1) overall, HIV-infection was not independently associated with changes in any CAD markers among the total study population, and (2) duration of cocaine use was significantly associated with increased coronary Agatston-scores, number of coronary plaques and SSS. However, these associations were more apparent in PLWH as compared to uninfected individuals. These findings imply that HIV-infection may indirectly promote CAD progression as other risk factors, such as duration of cocaine use may have a more prominent role in the acceleration of CAD in PLWH.

As life expectancy of PLWH irrespective of ART therapy is still shorter than that of the general population^1,2^, it is of utmost importance to better understand the possible causes of decreased life expectancy. Furthermore, as African Americans in the United States have been disproportionally affected by HIV-infection and drug abuse, it is crucial to understand whether and how HIV-infection exacerbates cardiovascular comorbidity among this population. Reports of the possible associations of HIV with CAD are inconsistent. While the Multicenter AIDS Cohort Study reported a significant association between HIV and an increased prevalence of noncalcified coronary plaque^3,4^, both a Baltimore prospective heart study and a Swedish prospective cohort study failed to identify a significant associated between HIV-infection and the presence of any plaque and noncalcified or mixed plaques^5,6^. Interestingly, the Swedish prospective cohort study demonstrated that HIV-infection was negatively associated with the total number of plaques and SSS^6^.

A possible reason for the conflicting results may be the fact that cross-sectional analyses are inherently limited in nature. There is a scarcity in longitudinal analyses on the effects of HIV-infection on CAD. Tarr et al. found similar results are our current investigation where HIV-infection was not associated with progression of CAD^24^. CAD is a dynamic process where noncalcified plaques progress over time into calcified plaques^25^. Cross-sectional studies only provide information regarding associations at a specific point in time and do not provide information on how risk factors may truly affect the course of a disease. Also, as PLWH are at an increased risk of using illegal substances^10,11^, the possible detrimental effects of drugs, such as cocaine use, potentially have a more prominent impact on the lives of these individuals. Lai et al. has found that in PLWH long-term cocaine use (defined as use for over 15 years) significantly increased the odds of significant stenosis^7^. Lai and colleges also found that chronic cocaine users had an increased prevalence ratio to have any coronary stenosis or Agatston-score when corrected for multiple risk factors^5^. However, among acute coronary syndrome patients, Bamberg et al. found no association between plaque markers and cocaine use. However, they were at increased risk of acute coronary syndrome as compared to matched individuals^8,26^. Similar results were found by Chang and colleges among acute coronary syndrome patients, where cocaine-use was not associated with coronary stenosis or calcifications^9^.

As compared to previous investigations our longitudinal assessment allows a unique opportunity to base our inferences on longitudinal changes in risk factors and CAD outcomes, rather than cross-sectional analyses. It seems that there is a linear relationship between the years of cocaine use and Agatston-score, the number of coronary plaques and also SSS. However this relationship is only apparent in PLWH. Our results may provide an explanation to why PLWH have accelerated CAD progression and why previous investigations are contradictory. It seems that while HIV-infection does not have a direct adverse effect on Agatston-score, the number of plaques or SSS, it may amplify the effects illicit drugs, such as cocaine. Therefore, cocaine use may have a prominent role in the development and acceleration of CAD interpedently from other risk factors in this patient population. While we have effective therapies for cardiovascular risk such as statins, the only way to overcome the negative effects of cocaine use is through abstinence. As PLWH report higher rates of substance use, it is of utmost importance to promote and achieve abstinence from illicit drugs.

Our study has several limitations. First, all our study subjects were African Americans. While racial/ethnic homogeneity could be considered a strength, the findings derived from this study population might not be generalizable to other racial and ethnic groups without caution. Furthermore, due to the longitudinal nature of our analysis, we did not assess the effect of risk factors on noncalcified, mixed or calcified plaque proportions. Since CAD is a dynamic process where noncalcified plaque progress into calcified plaques, accurate inferences regarding longitudinal compositional change can only be done using volumetric analysis. Also, only PLWH took statins, therefore we were not able to assess the effects of statin in uninfected individuals. Furthermore, our investigation only looked at cocaine use among potential illicit drugs. As illegal substance use is more common in this population, other illicit drugs may also have potentially affected our observed results.

In conclusion, PLWH are not destined to have worse CAD, as HIV-infection is not independently associated with any marker of CAD. However, cardiovascular risk factors and cocaine use may have a more profound effect on CAD progression in those with HIV-infection. Therefore, more aggressive management of cardiovascular risk factors and illicit drug use is needed. Furthermore, more strict normal values and earlier initiation of preventive therapies might be needed in these patients to achieve similar outcomes.

## Supplementary Information

Supplementary Information.

## Data Availability

The study involves delicate PII and PHI (HIV infection information), therefore may only be handled by individuals involved in the study, as the participants did not provide consent to provide their data to other parties.

## Abbreviations

ART: Antiretroviral therapy
ASCVD: Atherosclerotic cardiovascular disease
CAD: Coronary artery disease
CTA: CT angiography
HIV: Human immunodeficiency virus
hsCRP: High‐sensitivity C‐reactive protein
NRTI: Nucleoside reverse-transcriptase inhibitor
NNRTI: Non-nucleoside reverse-transcriptase inhibitor
PI: Protease inhibitor
PLWH: People living with HIV
SSS: Segment stenosis score

## Code availability

Analysis code may be provided upon reasonable request made to the corresponding author.
