# Supplementary material for: HIV indirectly accelerates coronary artery disease by promoting the effects of risk factors: longitudinal observational study
Source: Sci Rep. 2021 Nov 30;11:23110. doi: 10.1038/s41598-021-02556-w (PMC8632934; doi:10.1038/s41598-021-02556-w)
Supplement: Supplementary file 1 — Supplementary Information. [file 41598_2021_2556_MOESM1_ESM.pdf]

# SUPPLEMENTARY MATERIAL

# HIV indirectly accelerates coronary artery disease by promoting the effects of risk factors - longitudinal observational study

Márton Kolossváry, MD, PhD; David Celentano, ScD; Gary Gerstenblith, MD; David A Bluemke, MD; Raul N. Mandler, MD; Elliot K. Fishman, MD; Sandeepan Bhatia, MD; Shaoguang Chen, MS; Shenghan Lai, MPH; Hong Lai, PhD

## Supplemental text

| | |
|---|---|
| Supplemental methods | p2 |
| Supplemental tables | p5 |
| Supplemental references | p10 |

## SUPPLEMENTAL METHODS

### Definitions of demographic characteristics

At the time of each CT, patients underwent a detailed interview to obtain information about medical history and risk factors, including alcohol consumption, cocaine use and cigarette smoking. Regarding cardiovascular risk factors, hypertension was defined as systolic blood pressure >140 mmHg and/or diastolic blood pressure >90 mmHg or antihypertensive medication use verified by medical records. Diabetes was defined as a fasting plasma glucose ≥126 mg/dL or antidiabetic medication use verified by medical records. Positive family history for CAD was considered positive if first-degree relatives had a clinical diagnosis of significant CAD. A physical examination was performed, and anthropometrics were recorded. Routine clinical laboratory blood chemistry tests were conducted. The following laboratory tests were performed: total cholesterol; triglycerides; high-density lipoprotein; low-density lipoprotein glucose and high-sensitivity C-reactive protein.

### Native and coronary CTA acquisition protocols

Protocols were published earlier.[1,2] The participants' studies were performed at the Johns Hopkins Outpatient Center. Imaging was performed on a Sensation 64-slice Cardiac scanner (Siemens Medical Solutions, Erlangen, Germany) from May 2004 through June 2009 and a Siemens 128-slice dual-source CT scanner (Somatom Definition FLASH; Siemens Healthcare, Forchheim, Germany) from July 2009 through February 2015. Non-contrast examinations were performed using 120kVp with a slice thickness of 3 mm. For coronary CTA depending on patient size, 100 or 120 kVp was used with a rotation time of 0.33 seconds and a collimation of 64x0.6 mm for the 64-slice scanner and a rotation time of 0.28 seconds and a collimation of 128x0.6 mm for the 128-slice scanner. Scan data were reconstructed to a 512x512 matrix using a slice thickness of 0.75 mm at 0.4 mm increments for the 64-slice

scanner and 0.5 mm increments for the 128-slice scanner. The scan protocol used between 80mL and 100 mL of an iodixanol based contrast agent (Vispaque-320; GE Medical Systems, GE Healthcare Ireland, Cork, Ireland) injected at 5 to 6 cc/sec with test bolus data used to calculate the peak contrast enhancement to determine correct scan delay. All image data were transferred to a dedicated workstation for analysis (Syngo.via; Siemens Medical Solutions, Malvern, PA). All images were randomly interpreted by a level-3 certified specialist in cardiac CT imaging with 5 years of experience and blinded to all clinical data.

**Statistical analysis**

Continuous variables are presented as means and standard deviations, while categorical variables are shown as frequencies and percentages. Continuous variables were compared using the t-test or the paired t-test as appropriate, while categorical parameters were compared using the Fisher's-test or McNemar's-test as appropriate.

We used linear mixed models to analyze our longitudinal data. We first assessed the need to include a random intercept at a patient level. Next we evaluated the need to include a random slope term per-patient. For the random slope we used the time difference between the baseline and the follow-up scans.[3] We used the likelihood ratio test to assess the potential significant deterioration in fit if we were to remove one or both random terms when we only had a fixed intercept term. Our results show the need for both random slope and intercept for all outcomes (supplemental tables 5-7*)*. We then ran univariate models by adding each predictor, time difference between the two scans and their interaction as fixed effects. Restricted maximum likelihood was used to fit the models. We assessed the statistical significance of the fixed terms using Satterthwaite's method. Furthermore, we calculated the confidence intervals by computing a likelihood profile and finding the appropriate cutoffs based on the likelihood ratio test. If either the main effect or the interaction with time had a p value smaller than 0.10,

we included the parameter in the multivariate model. For the multivariate model, we included all significant parameters, time between the CT scans, and their interactions with time into the models. Linear mixed models were built using the lme4 (v.1.1-21) package.[4] P values were calculated using the lmerTest (v.3.1-0) package.[5] All calculations were performed in the R (v. 3.6.1) environment [6].

## SUPPLEMENTAL TABLES

**Supplemental table 1.** Annual progression rates of coronary artery disease parameters stratified by HIV-serostatus.

| Coronary artery disease parameters | Annual progression rates | | |
|---|---|---|---|
| | **HIV-infected** | **HIV-uninfected** | **p** |
| **Clinical characteristics** | | | |
| Agatston-score (unit/year) | 10.83±25.13 | 7.19±17.84 | 0.174 |
| Number of plaques (unit/year) | 0.23±0.28 | 0.33±0.47 | 0.106 |
| Segment Stenosis Score (unit/year) | 0.53±0.84 | 0.54±1.29 | 0.961 |

Data reported as average and standard deviation.

**Supplemental table 2.** Demographic, behavioral, clinical, laboratory and coronary artery disease characteristics at baseline and follow-up stratified by cocaine use.

| Characteristic | Cocaine users (n=174) | | | Cocaine non-users (n=126) | | | Cocaine users vs. Cocaine non-users | |
|---|---|---|---|---|---|---|---|---|
| | Baseline | Follow-up | p | Baseline | Follow-up | p | Baseline p | Follow-up p |
| **Anthropometrics** | | | | | | | | |
| Age (year) | 48.6 ± 6.2 | 53.1 ± 6.5 | <0.0001 | 47.1 ± 8.4 | 50.3 ± 8.1 | <0.0001 | 0.083 | 0.001 |
| Male sex (n, %) | 126 (72.4%) | 126 (72.4%) | 1.000 | 84 (66.7%) | 84 (66.7%) | 1.000 | 0.308 | 0.308 |
| BMI (kg/m$^2$) | 26.4 ± 5.5 | 26.9 ± 5.7 | 0.075 | 26.8 ± 4.9 | 27.0 ± 5.3 | 0.511 | 0.509 | 0.900 |
| Follow-up time (years) | - | 4.5 ± 2.6 | - | - | 3.2 ± 1.6 | - | - | <0.0001 |
| **Cardiovascular risk factors** | | | | | | | | |
| ASCVD risk (%) | 7.7 ± 5.9 | 10.9 ± 7.4 | <0.0001 | 7.4 ± 8.7 | 9.2 ± 8.3 | 0.017 | 0.796 | 0.085 |
| Hypertension (n, %) | 25 (14.4%) | 49 (28.2%) | <0.0001 | 18 (14.3%) | 40 (31.7%) | <0.0001 | 1.000 | 0.524 |
| Diabetes (n, %) | 6 (3.4%) | 13 (7.5%) | 0.023 | 3 (2.4%) | 8 (6.3%) | 0.074 | 0.739 | 0.820 |
| Positive family history (n, %) | 45 (25.9%) | 58 (33.3%) | <0.001 | 34 (27.0%) | 45 (35.7%) | 0.003 | 0.894 | 0.712 |
| Report of cigarette use (n, %) | 160 (92.0%) | 162 (93.1%) | 0.48 | 88 (69.8%) | 89 (70.6%) | 1.000 | <0.0001 | <0.0001 |
| Report of alcohol use (n, %) | 161 (92.5%) | 168 (96.6%) | 0.023 | 94 (74.6%) | 103 (81.7%) | 0.008 | <0.0001 | <0.0001 |
| Statin users (n, %) | 15 (8.6%) | 21 (12.1%) | 0.041 | 10 (7.9%) | 10 (7.9%) | 1.000 | 1.000 | 0.337 |
| **Lipid profiles and laboratory results** | | | | | | | | |
| Total cholesterol (mg/dL) | 174.5 ± 41.1 | 171.0 ± 40.7 | 0.156 | 173.2 ± 37.0 | 174.3 ± 36.4 | 0.637 | 0.776 | 0.468 |
| LDL-C (mg/dL) | 94.3 ± 36.9 | 86.5 ± 31.9 | 0.002 | 96.1 ± 34.1 | 96.8 ± 31.2 | 0.997 | 0.659 | 0.007 |
| HDL-C (mg/dL) | 55.9 ± 18.2 | 59.1 ± 23.2 | 0.01 | 50.6 ± 18.4 | 52.6 ± 18.6 | 0.075 | 0.015 | 0.009 |
| Triglycerides (mg/dL) | 125.3 ± 79.2 | 130.7 ± 86.1 | 0.575 | 124.2 ± 75.2 | 132.9 ± 87.0 | 0.232 | 0.901 | 0.829 |
| Fasting glucose (mg/dL) | 88.4 ± 15.8 | 91.9 ± 21.2 | 0.021 | 91.7 ± 40.0 | 100.1 ± 62.4 | 0.068 | 0.388 | 0.167 |
| hsCRP (mg/dL) | 3.4 ± 5.2 | 5.4 ± 9.9 | 0.005 | 3.1 ± 4.5 | 4.2 ± 10.6 | 0.253 | 0.596 | 0.338 |
| **HIV associated factors** | | | | | | | | |
| HIV-infected (n, %) | 124 (71.3%) | 124 (71.3%) | 1.000 | 102 (81.0%) | 102 (81.0%) | 1.000 | 0.059 | 0.059 |
| Time since HIV diagnosis (year) | 21.5 ± 8.8 | 26.2 ± 9.6 | <0.0001 | 22.0 ± 8.8 | 25.0 ± 9.0 | <0.0001 | 0.692 | 0.310 |
| Antiretroviral therapy users (n, %) | 106 (89.1%) | 110 (94.0%) | 0.134 | 94 (90.4%) | 97 (98.0%) | 0.134 | 0.827 | 0.184 |
| NRTI users (n, %) | 101 (84.9%) | 104 (88.9%) | 0.248 | 89 (85.6%) | 93 (93.9%) | 0.074 | 1.000 | 0.233 |
| Duration of NRTI use (year) | 6.2 ± 4.9 | 9.4 ± 6.3 | <0.0001 | 5.1 ± 5.6 | 7.1 ± 5.9 | <0.0001 | 0.163 | 0.008 |
| NNRTI users (n, %) | 46 (38.7%) | 51 (43.6%) | 0.074 | 54 (51.9%) | 59 (59.6%) | 0.074 | 0.059 | 0.021 |
| Duration of NNRTI use (year) | 4.9 ± 4.0 | 7.0 ± 5.1 | <0.0001 | 5.2 ± 5.3 | 6.9 ± 5.8 | <0.0001 | 0.740 | 0.922 |
| PI users (n, %) | 90 (75.6%) | 97 (82.9%) | 0.023 | 68 (65.4%) | 69 (69.7%) | 0.480 | 0.105 | 0.024 |
| Duration of PI use (year) | 5.8 ± 5.4 | 8.6 ± 6.6 | <0.0001 | 6.4 ± 8.9 | 8.4 ± 9.1 | <0.0001 | 0.603 | 0.889 |
| **Visual characteristics of coronary artery disease** | | | | | | | | |
| Agatston-score (unit) | 71.0 ± 219.1 | 130.6 ± 315.1 | <0.0001 | 23.7 ± 69.2 | 41.8 ± 122.7 | 0.003 | 0.008 | <0.001 |
| Number of plaques (unit) | 2.2 ± 2.2 | 3.1 ± 2.3 | <0.0001 | 1.9 ± 1.6 | 2.5 ± 1.6 | <0.0001 | 0.268 | 0.01 |
| Segment Stenosis Score (unit) | 3.2 ± 3.6 | 5.4 ± 5.4 | <0.0001 | 2.9 ± 3.2 | 4.0 ± 3.6 | <0.0001 | 0.420 | 0.122 |

Average and standard deviation for continuous variables, frequencies and proportion (%) for categorical variables are reported. P-values are based on t-test or paired t-test as appropriate or chi-square test or McNemar-test as appropriate.

Abbreviations: ASCVD risk: cardiovascular risk defined by the ACC/AHA Guideline on the Assessment of Cardiovascular Risk; BMI: body mass index (kg/m2); LDL-C: low density lipoprotein cholesterol; HDL-C: high density lipoprotein cholesterol; HIV: human immunodeficiency virus; hsCRP: high-sensitivity C-reactive protein; NRTI: nucleoside reverse-transcriptase inhibitors; NNRTI: non-nucleoside reverse-transcriptase inhibitors; PI: protease inhibitors.

**Supplemental table 3.** Predictors of coronary artery disease outcomes among cocaine-users

| **Outcome** | **Predictor** | **Univariate models** | | | | | | **Multivariate models** | | | | | |
|---|---|---|---|---|---|---|---|---|---|---|---|---|---|
| | | **Overall effect on outcome** | | | **Effect on progression rate** | | | **Overall effect on outcome** | | | **Effect on progression rate** | | |
| | | β | 95% CI | p | β | 95% CI | p | β | 95% CI | p | β | 95% CI | p |
| **Agatston-score (unit)** | HIV* | 39.05 | [-32.85-110.96] | 0.289 | 5.39 | [-2.75-13.53] | 0.196 | 26.73 | [-46.21-99.57] | 0.471 | 1.55 | [-5.90-9.00] | 0.681 |
| | ASCVD (%) | 0.11 | [-1.71-2.01] | 0.906 | **0.98** | **[0.43-1.52]** | **<0.001** | 0.06 | [-1.58-1.80] | 0.944 | **0.73** | **[0.21-1.25]** | **0.006** |
| | Positive family history | 16.53 | [-23.04-56.10] | 0.415 | **8.73** | **[ 0.68-16.77]** | **0.035** | 25.62 | [-11.44-62.35] | 0.171 | 3.52 | [-4.01-11.01] | 0.356 |
| | Statin use | **101.51** | **[ 50.18-152.65]** | **<0.001** | **11.92** | **[ 0.60-23.29]** | **0.041** | **95.91** | **[45.47-145.66]** | **<0.0001** | 6.98 | [-4.53-18.58] | 0.235 |
| | hsCRP (mg/dL) | 0.22 | [-1.08-1.53] | 0.742 | -0.11 | [-0.71-0.50] | 0.731 | | | | | | |
| **Number of coronary plaques (unit)** | HIV* | 0.07 | [-0.65-0.80] | 0.843 | 0.00 | [-0.08-0.08] | 0.995 | -0.05 | [-0.73-0.63] | 0.882 | -0.02 | [-0.11-0.06] | 0.604 |
| | ASCVD (%) | **0.06** | **[0.02-0.09]** | **<0.001** | **-0.01** | **[-0.01-0.00]** | **0.064** | **0.06** | **[0.03-0.09]** | **<0.0001** | 0.00 | [-0.01-0.00] | 0.203 |
| | Positive family history | **0.59** | **[0.08-1.10]** | **0.024** | -0.07 | [-0.14-0.01] | 0.081 | **0.61** | **[0.12-1.09]** | **0.015** | **-0.08** | **[-0.16-0.00]** | **0.039** |
| | Statin use | **1.16** | **[0.42-1.90]** | **0.002** | -0.05 | [-0.16-0.05] | 0.276 | **1.05** | **[0.34-1.76]** | **0.004** | -0.03 | [-0.14-0.07] | 0.540 |
| | hsCRP (mg/dL) | **0.03** | **[0.01-0.06]** | **0.003** | **-0.01** | **[-0.01-0.00]** | **0.010** | **0.04** | **[0.02-0.06]** | **<0.0001** | **-0.01** | **[-0.01-0.00]** | **0.003** |
| **Segment Stenosis Score (unit)** | HIV* | 0.16 | [-1.17-1.49] | 0.814 | 0.13 | [-0.11-0.38] | 0.280 | -0.13 | [-1.32-1.05] | 0.825 | 0.08 | [-0.16-0.32] | 0.487 |
| | ASCVD (%) | **0.12** | **[0.05-0.20]** | **<0.001** | 0.01 | [-0.01-0.02] | 0.423 | **0.12** | **[0.05-0.18]** | **<0.0001** | 0.01 | [-0.01-0.02] | 0.398 |
| | Positive family history | 0.94 | [-0.19-2.07] | 0.104 | -0.01 | [-0.24-0.22] | 0.958 | | | | | | |
| | Statin use | **3.34** | **[1.73-4.93]** | **<0.0001** | 0.11 | [-0.19-0.40] | 0.478 | **3.21** | **[1.73-4.68]** | **<0.0001** | 0.05 | [-0.26-0.36] | 0.755 |
| | hsCRP (mg/dL) | **0.13** | **[0.08-0.19]** | **<0.0001** | **-0.02** | **[-0.03-0.00]** | **0.045** | **0.15** | **[0.10-0.20]** | **<0.0001** | **-0.02** | **[-0.04-0.00]** | **0.013** |

*: HIV was forced into the multivariate model. Parameters showing an association with a given outcome with p<0.10 were included in the multivariate model. Bold indicates statistical significance.

Abbreviations: β: unstandardized beta coefficients from linear mixed model; ASCVD risk: cardiovascular risk defined by the ACC/AHA Guideline on the Assessment of Cardiovascular Risk; CI: confidence interval; HIV: human immunodeficiency virus; hsCRP: high-sensitivity C-reactive protein.

**Supplemental table 4.** Predictors of coronary artery disease outcomes among cocaine non-users

| Outcome | Predictor | Univariate models | | | | | | Multivariate models | | | | | |
|---|---|---|---|---|---|---|---|---|---|---|---|---|---|
| | | Overall effect on outcome | | | Effect on progression rate | | | Overall effect on outcome | | | Effect on progression rate | | |
| | | β | 95% CI | p | β | 95% CI | p | β | 95% CI | p | β | 95% CI | p |
| **Agatston-score (unit)** | HIV* | 6.28 | [-24.57-37.12] | 0.691 | 2.88 | [-6.57-12.33] | 0.552 | 0.98 | [-29.28-31.34] | 0.949 | -2.73 | [-11.23-5.78] | 0.527 |
| | ASCVD (%) | **1.23** | **[0.29-2.17]** | **0.011** | **1.04** | **[0.62-1.46]** | **<0.0001** | **1.21** | **[0.25-2.16]** | **0.012** | **1.07** | **[0.64-1.50]** | **<0.0001** |
| | Positive family history | 1.96 | [-19.65-23.27] | 0.856 | 6.04 | [-1.96-13.99] | 0.140 | | | | | | |
| | Statin use | -14.56 | [-59.32-30.20] | 0.525 | -2.03 | [-15.77-11.71] | 0.773 | | | | | | |
| | hsCRP (mg/dL) | 0.23 | [-1.21-1.66] | 0.759 | -0.20 | [-0.87-0.48] | 0.573 | | | | | | |
| **Number of coronary plaques (unit)** | HIV* | 0.20 | [-0.52-0.91] | 0.592 | 0.07 | [-0.03-0.17] | 0.148 | 0.11 | [-0.60-0.81] | 0.767 | 0.08 | [-0.02-0.18] | 0.108 |
| | ASCVD (%) | **0.02** | **[0.00-0.04]** | **0.020** | 0.00 | [-0.01-0.00] | 0.512 | **0.02** | **[0.00-0.04]** | **0.016** | 0.00 | [-0.01-0.00] | 0.329 |
| | Positive family history | 0.21 | [-0.19-0.62] | 0.304 | 0.03 | [-0.08-0.13] | 0.594 | | | | | | |
| | Statin use | 0.85 | [-0.18-1.88] | 0.109 | 0.09 | [-0.06-0.23] | 0.258 | | | | | | |
| | hsCRP (mg/dL) | 0.01 | [-0.01-0.04] | 0.244 | 0.00 | [-0.01-0.01] | 0.512 | | | | | | |
| **Segment Stenosis Score (unit)** | HIV* | 0.60 | [-0.88-2.09] | 0.426 | **0.24** | **[0.01-0.47]** | **0.045** | 0.40 | [-1.02-1.83] | 0.577 | 0.10 | [-0.11-0.30] | 0.343 |
| | ASCVD (%) | 0.03 | [-0.01-0.07] | 0.107 | **0.02** | **[0.00-0.03]** | **0.008** | 0.04 | [0.00-0.08] | 0.061 | **0.01** | **[0.00-0.03]** | **0.036** |
| | Positive family history | **1.01** | **[0.07-1.96]** | **0.037** | 0.12 | [-0.12-0.36] | 0.319 | **0.99** | **[0.10-1.89]** | **0.030** | 0.04 | [-0.18-0.26] | 0.714 |
| | Statin use | 0.62 | [-1.56-2.80] | 0.579 | **0.56** | **[0.21-0.90]** | **0.001** | 0.43 | [-1.63-2.50] | 0.680 | **0.51** | **[0.19-0.84]** | **0.002** |
| | hsCRP (mg/dL) | 0.02 | [-0.03-0.07] | 0.435 | 0.01 | [-0.01-0.03] | 0.332 | | | | | | |

*: HIV was forced into the multivariate model. Parameters showing an association with a given outcome with p<0.10 were included in the multivariate model. Bold indicates statistical significance.

Abbreviations: β: unstandardized beta coefficients from linear mixed model; ASCVD risk: cardiovascular risk defined by the ACC/AHA Guideline on the Assessment of Cardiovascular Risk; CI: confidence interval; HIV: human immunodeficiency virus; hsCRP: high-sensitivity C-reactive protein

**Supplemental table 5.** Model fit assessment regarding random effect terms for outcome: Ca-score.

| Removal of term | Log Likelihood | AIC | –ΔLog Likelihood | Degrees of freedom | p |
|---|---|---|---|---|---|
| – | -3729.4 | 7466.7 | – | – | – |
| Random slope | -3891.0 | 7788.0 | 161.6 | 1 | $< 10^{-15}$ |
| Random intercept | -4056.7 | 8119.4 | 327.3 | 1 | $< 10^{-15}$ |
| Random intercept and slope | -4083.7 | 8171.4 | 354.3 | 2 | $< 10^{-15}$ |

**Supplemental table 6.** Model fit assessment regarding random effect terms for outcome: Number of coronary plaques.

| Removal of term | Log Likelihood | AIC | –ΔLog Likelihood | Degrees of freedom | p |
|---|---|---|---|---|---|
| – | -1104.8 | 2217.5 | – | – | – |
| Random slope | -1117.7 | 2241.5 | 12.9 | 1 | $< 10^{-7}$ |
| Random intercept | -1285.9 | 2577.8 | 181.1 | 1 | $< 10^{-15}$ |
| Random intercept and slope | -1289.0 | 2582.1 | 184.2 | 2 | $< 10^{-15}$ |

**Supplemental table 7.** Model fit assessment regarding random effect terms for outcome: Segment stenosis score.

| Removal of term | Log Likelihood | AIC | –ΔLog Likelihood | Degrees of freedom | p |
|---|---|---|---|---|---|
| – | -1104.8 | 2217.5 | – | – | – |
| Random slope | -1117.7 | 2241.5 | 12.9 | 1 | $< 10^{-7}$ |
| Random intercept | -1285.9 | 2577.8 | 181.1 | 1 | $< 10^{-15}$ |
| Random intercept and slope | -1289.0 | 2582.1 | 184.2 | 2 | $< 10^{-15}$ |

**SUPPLEMENTAL REFERECES**


1 Lai, S. *et al.* Long-term cocaine use and antiretroviral therapy are associated with silent coronary artery disease in African Americans with HIV infection who have no cardiovascular symptoms. *Clin Infect Dis* **46**, 600-610, doi:10.1086/526782 (2008).

2 Lai, H. *et al.* HIV Infection Itself May Not Be Associated With Subclinical Coronary Artery Disease Among African Americans Without Cardiovascular Symptoms. *J Am Heart Assoc* **5**, e002529, doi:10.1161/JAHA.115.002529 (2016).

3 Morrell, C. H., Brant, L. J. & Ferrucci, L. Model choice can obscure results in longitudinal studies. *J Gerontol A Biol Sci Med Sci* **64**, 215-222, doi:10.1093/gerona/gln024 (2009).

4 Bates, D., Mächler, M., Bolker, B. & Walker, S. Fitting Linear Mixed-Effects Models Using lme4. *2015* **67**, 48, doi:10.18637/jss.v067.i01 (2015).

5 Kuznetsova, A., Brockhoff, P. B. & Christensen, R. H. B. lmerTest Package: Tests in Linear Mixed Effects Models. *2017* **82**, 26, doi:10.18637/jss.v082.i13 (2017).

6 R: A Language and Environment for Statistical Computing v. 3.5.2 (R Foundation for Statistical Computing, 2018).
